# Motivation to succeed is not enough: motivated students need to know how to plan/organize their steps on their way to success

**DOI:** 10.3389/fpsyg.2023.1119409

**Published:** 2023-06-13

**Authors:** Elena Lisá, Lenka Sokolová, Paulína Jablonická, Lea Kardelisová

**Affiliations:** Institute of Applied Psychology, Faculty of Social and Economic Sciences, Comenius University in Bratislava, Bratislava, Slovakia

**Keywords:** motivation, academic achievement, employability, self-efficacy, planning and organizing, learning strategy

## Abstract

The study is based on dispositional (career motivation) and social-cognitive (generalized self-efficacy) theories of personality, further on the expectancy-value theory of achievement motivation and future time perspective theory (task value, time, and study environment). The study aimed to explain the mechanism of the prediction relationship between motivation and students’ performance. It was assumed that skills of planning and organizing (operationalized as generalized self-efficacy and learning strategies) mediate the prediction of motivation (career motivation and task value) on students’ success (operationalized as academic achievement and employability). In two studies (*N* = 313, *N* = 219), the hypotheses of the mediation models were supported by structural equation modeling. Generally, the skills of organizing/planning fully mediated the students’ performance, measured as academic achievement and employability (number of employers). The results show the importance of combining dispositional motivation characteristics with dynamic planning skills on the way to students’ success. Traditional psychological predictors of performance, like general mental ability and conscientiousness, were not controlled. Higher education institutions could support motivated students on their way to success by teaching them how to plan and organize specific steps on their way to success.

## 1. Introduction

General psychological predictors of performance include a combination of general mental ability, conscientiousness, and experience (Salgado, 2016). By combining psychological predictors, we can predict more than 60% of performance, corresponding to individual differences. The psychological predictors of job performance most frequently assessed are the general mental ability, skills, conscientiousness, examples of work performance and integrity (Hough and Furnham, 2003; Scroggins et al., 2009; Vinchur and Koppes Bryan, 2012; Salgado, 2016).

Lisá (2018) focused on examining student academic performance predictors. A significant relationship was found between the performance of the students and their intelligence in primary schools. This assumption was based on several previous studies (Colom et al., 2007; Chamorro-Premuzic and Furnham, 2008; Krumm et al., 2008). However, in the predictors of academic success among university students, intelligence no longer plays a significant role. Furnham et al. (2003) presented that intelligence is not an essential predictor of the academic success of university students. Lisá (2018) confirmed that intelligence is a significant predictor of academic success only among students in primary and secondary schools, not in universities. Kriegbaum et al. (2015) demonstrated that efficacy is a stronger predictor of academic success than intelligence, thus confirming the conclusion that the importance of self-efficacy and motivation for predicting performance increases with age.

For a long time, the importance of cognitive factors was emphasized in predicting school performance. Still, many researchers have begun to realize that the variability of factors influencing academic success is considerable and that it is necessary to investigate a broader spectrum of factors (Parker et al., 2004; Medveďová and Lisá, 2010). Travers’s (1949) findings are more than half a century old. Still, his studies have already demonstrated the suitability of combining cognitive and noncognitive factors in predicting academic success. At the same time, he stated that the contribution of non-intellectual factors is not sufficiently emphasized. Today’s situation is not very different from the one 73 years ago.

Two current studies aim to demonstrate the importance of predictors of a noncognitive nature, especially the combination of motivation with the skill to plan and organize. The assumption is that motivation, a predictor of success in university students, will be mediated by the skill to organize and plan. The goal is to explain the mechanism of the predictive relationship between motivation and performance and show its greater potential. Therefore, a mediator of dynamic personality features (self-efficacy or learning strategy) was included, and structural equation modeling was applied. Two studies with different operationalizations of the investigated variables and research samples were implemented.

The first research study will verify the predictive relationship between career motivation and employability in university students, mediated by generalized self-efficacy. Career motivation (Fugate and Kinicki, 2008) is considered a predictor. Generalized self-efficacy (Bandura, 1977) is considered a noncognitive mediator/predictor of student success in terms of planning and organizing one’s steps on the way to the goal. Employability is operationalized as the number of past and recent employers. It is considered the measure of the students’ success.

The second research study will investigate the prediction of the academic achievement of higher education students by task value as a motivational aspect of their studies and with the organization of time and study environment as a mediator. Academic achievement is operationalized as the weighted average of self-reported grades in the previous semester and general studies - for this study, it is considered the measure of students’ success in higher education. Task value is defined as a motivational predictor of academic achievement (Liem et al., 2008; Li et al., 2021), while the organization of time and the study environment functions as a mediator.

## 2. Definition of variables

### 2.1. Motivation to succeed

Motivation is usually an additional predictor of success in life/school and work. Furnham (2005) mentioned motivation among the six predictors of job success. People can be motivated by various factors such as success, power, money, promotion, etc. When evaluating motivation, three questions are essential: Is it healthy? Is it realistic? Is it temporary or long-term? Therefore, ambition and the need for performance and success are necessary for good performance. Because they are the engine and the guide of work behavior, their lack indicates a waste of talent. According to a meta-analysis, intelligence and motivation predict school performance, with unique and common shares (Kriegbaum et al., 2018). Many studies in the past have shown that motivation is a significant predictor of student academic success (Caldwell and Obasi, 2010; Dogan, 2015).

Different definitions of motivation are related to success at work or in studies. Popular is the study of achievement motivation (Furnham, 2005; Caldwell and Obasi, 2010), motivation within self-determination theory (Wo et al., 2016), motivation to learn (Tentama and Arridha, 2020), and career motivation (Fugate and Kinicki, 2008).

#### 2.1.1. Career motivation

Career motivation is essential for employees’ continuous education and employability. Employees with a high degree of dispositional career motivation plan their futures and take advantage of various training and learning opportunities. They are also characterized by a willingness to change to meet the situational demands of their work environment and complete their set goal (Fugate and Kinicki, 2008). Career motivation is based on the concepts of motivational control (Kanfer and Heggestad, 1997) and learning goal orientation (Dweck and Leggett, 1988). By setting their own goals, employees with high motivational control are more motivated at work and exert effort to complete their goals despite challenges and changes in their work environment. They can also resist more when frustrated or bored (Kanfer and Heggestad, 1997).

#### 2.1.2. Task value

Task value and intrinsic goals orientation are motivational beliefs related to subjective motivational values ascribed to educational content and outcomes (Stegers-Jager et al., 2012). Task value refers to a student’s evaluation of how interesting, important, or useful the task or educational content is in general, and high task value should lead to more engagement in learning (Pintrich et al., 1991). Eccles and Wigfield differentiated four areas of task values: intrinsic value, utility value, attainment value, and cost (Eccles and Wigfield, 1995, 2002). The concept of task value is based on the expectancy × value theory of achievement motivation and future time perspective theory (Husman et al., 2004). Some authors highlight the connection between task value and self-efficacy, as task value refers to the importance or usefulness of the task, and self-efficacy refers to an individual’s belief in his ability to perform the task (Bong, 2001; Neuville et al., 2007).

### 2.2. Ability to plan

#### 2.2.1. Self-efficacy

Self-efficacy refers to personal beliefs or confidence in performing effectively specified tasks. It affects behavior and motivation. The social cognitive theory states four primary sources of efficacy expectations: previous performance achievements, vicarious experiences, verbal persuasion, and physiological states (Bandura, 1977). These sources show the dynamic nature of self-efficacy and enable one to plan and organize steps to the goal. “Expectations of personal efficacy do not operate as dispositional determinants independently of contextual factors.” (Bandura, 1977, p. 203). Therefore, it is necessary for a subject to identify the circumstance and determine the required behavior. Recent research shows that the higher self-efficacy, the higher planning skills, and the tendency to physical activity (Koring et al., 2012), the higher planning of post-training activities (Bruyere et al., 2022). Self-efficacy beliefs are rooted in support of a sense of confidence provided by the caregiver; as children develop positive attitudes, they receive support from adults’ tolerant behavior (Bandura, 1997). Generalized self-efficacy is affected by early memories of warmth and safety (Yilmaz Bingöl, 2018). Research studies show that other dynamic variables can influence the overall self-efficacy score.

#### 2.2.2. Learning strategies

Learning strategies can be described as learner behaviors that are intended to influence how the learner processes the educational content, knowledge, skills, etc. (Mayer, 1988). They are related to cognitive styles and strategies, but also to the organizational and motivational aspects of learning. Pintrich and De Groot (1990) introduced the concept of self-regulated learning, in which students are active participants in their learning, they plan and monitor their learning behaviors. This monitoring and controlling of cognitive performance refer to situations before, during, and after a learning episode (Li et al., 2018). As such self-regulated learning combines three components: metacognition, motivation, and behaviors, i.e., actual study strategies; and it correlates positively with academic achievement and success (Pintrich and De Groot, 1990; Credé and Phillips, 2011; Hilpert et al., 2013; Stark, 2019). Metacognition and motivation lead to appropriate learning strategies, which positively impact academic performance (Credé and Phillips, 2011; Li et al., 2018). A similar relationship was found between self-efficacy and academic achievement (Yip, 2012, 2021), meaning that self-regulated learning strategies are connected with self-efficacy and motivational variables.

### 2.3. Students’ success

#### 2.3.1. Employability

Employability is generally understood as “an individual’s chance of a job in the internal and/or external labor market” (Forrier et al., 2015, p. 1). There are various definitions of employability. Dispositional employability expresses personal adaptability, which is increasingly important to employees and employers in today’s dynamic work environment (Fugate and Kinicki, 2008). Employability is also a set of competencies/skills (Lisá et al., 2019; Römgens et al., 2020; Anderson and Tomlinson, 2021). But it is also defined in terms of objective criteria, such as the presence of employment or the number of employers. Several researchers have tried to explain why some people are more employable than others. This question can be viewed from individual or contextual perspectives (Fugate et al., 2021).

#### 2.3.2. Academic performance

Traditionally, high school grades and scores on standardized tests (e.g., intelligence tests) are considered predictors of college or university persistence, academic performance, and success (Sulaiman and Mohezar, 2006; Friedman and Mandel, 2011; Sparkman et al., 2012; van der Zanden et al., 2018). However, according to more recent studies, these explain only a modest amount of variance in a student’s academic performance (Kuncel et al., 2004; Sparkman et al., 2012), and that is why researchers focus on nontraditional predictors of academic performance and success: study skills or social relationships (van der Zanden et al., 2018), emotional intelligence (Sparkman et al., 2012), personality variables (Mills and Blankstein, 2000), academic self-concept (Wouters et al., 2011), the level of anxiety (Křeménková et al., 2019) or motivation (Steinmayr and Spinath, 2009; van der Zanden et al., 2018).

## 3. Current state of knowledge

### 3.1. Motivation as a predictor of success in students

According to Steinmayr and Spinath (2009), different motivational constructs can contribute to the prediction of academic achievement and explain performance variance, which is not explained by general mental abilities like intelligence. According to some studies, motivational beliefs, including intrinsic goals for learning, self-efficacy, and task value, are related to both effective study strategies and consequently better academic performance (Pintrich, 1999; Stegers-Jager et al., 2012). According to Friedman and Mandel (2011), students’ needs for achievement and autonomy at the start of college or university education significantly predicted grades at the end of their first year. Motivation to learn predicts students’ employability, explaining up to 55.8 of its variability (Tentama and Arridha, 2020). Intrinsic motivation significantly predicted employability in people with epilepsy (Wo et al., 2016).

### 3.2. Motivation as a predictor of ability to plan

More empirical research studies confirm a positive association between self-efficacy and achievement motivation (Abbasianfard et al., 2010; Habibah et al., 2010; Mohamadi et al., 2014; Zhang et al., 2015; Mouloud and Elkader, 2016). There is a small to medium correlation between self-efficacy and achievement motivation (Jalal et al., 2016; Harahsheh, 2017; Liqin and Lesen, 2018), but also no significant correlation (Sharma, 2015; Zhang et al., 2015). Achievement Motivation and its dimensions (confidence in success, dominance, competitiveness, and independence) predict generalized self-efficacy and explain 46% of general variability in self-efficacy (Lisá, 2020). Confidence in success reflects a tendency to achieve success even when there are obstacles to overcome (Schuler and Prochaska, 2011). This phenomenon was described by Bandura (1993) as a critical behavioral strategy of highly efficient thinking. Career motivation is also a significant predictor of self-efficacy (Fugate et al., 2004; Deng et al., 2022), and it creates a higher sense of self-efficacy. Task value is often considered a predictor of academic achievement and is connected with effective study strategies (Pintrich et al., 1991; Bong, 2001; Neuville et al., 2007; Liem et al., 2008; Li et al., 2021). Intrinsic academic motivation is positively related to academic achievement, especially student self-concepts and task values, which appear to be strong predictors of academic achievement (Steinmayr et al., 2019).

### 3.3. Ability to plan as a predictor of performance/success and employability

As confirmed by meta-analyses of previous research (Robbins et al., 2004), students’ self-efficacy proved to be an essential predictor of their subsequent academic success (Gutiérrez and Tomás, 2019). The construct of self-efficacy conceptually relates to employability in several ways. Various authors have different opinions on the relationship between these two constructs. Some consider them equivalent (Daniels et al., 1998; Washington, 1999), others as two distinct and separate constructs (Berntson et al., 2008), and still others as related phenomena (Nauta et al., 2002). The findings show that the constructs of employability and self-efficacy reflect related but also separate attributes and qualities. Self-efficacy is connected to one’s perceived feeling and self-assessment of one’s ability to perform various tasks (Bandura, 1997; Berntson et al., 2008), while employability reflects the perceived possibility of obtaining employment and is closely related to multiple specific skills such as skills acquired through education and practice (Fugate et al., 2004). Current research confirms that self-efficacy is related (Ahmed et al., 2019), mediates (Liu et al., 2020; Zhong et al., 2020; Zhao et al., 2021) or predicts (Magagula et al., 2020) employability.

Current research also supports the hypothesis that differences in academic performance among higher education students are largely due to the way they learn, that is, their learning strategies (Watson et al., 2004; Credé and Phillips, 2011; Yip, 2012, 2021; Basila, 2014; van der Zanden et al., 2018). Mills and Blankstein (2000) reported an association between self-oriented perfectionism and self-efficacy for learning and performance, adaptive metacognitive and cognitive learning strategies, and effective resource management, which are components of self-regulated learning. According to the concept of self-regulated learning, motivation and learning strategies are interrelated and both have an impact on an individual’s learning outcomes and performance (Pintrich and De Groot, 1990; Credé and Phillips, 2011).

The above-mentioned findings are highly relevant in COVID and post-COVID education because long-term online and hybrid teaching and learning can lead to changes in study behavior among higher education students and affect their motivation to study (Marler et al., 2021). According to Stark (2019), students in online courses reported lower levels of motivation than students in face-to-face courses; however, the author found a strong correlation between motivation variables and course performance in online education. Similarly, Basila (2014) concluded that time management study strategies and motivation are important predictors of academic success in online courses. The predictors of academic achievement seem to be equivalent for online and hybrid or blended education: time management, elaboration, and rehearsal strategies (Broadbent, 2017). These pre-COVID findings may reflect the situation of distance education during the COVID-19 pandemic.

In both distance and face-to-face education, students need to manage and regulate their time and their study environments (Pintrich et al., 1991), which involves scheduling, planning, the effective use of study time, setting realistic goals, and the choice of appropriate study environment. Study management skills help students to reach their study goals; according to some findings they are even stronger predictors of first-semester academic performance than general aptitude (West and Sadoski, 2011). Time management behaviors in higher education students had also buffering effect on academic stress (Misra and McKean, 2000).

## 4. The purpose of the study

The purpose of the study is show that the motivation itself is not enough to reach the success, that there are active mediators that enable the prediction to be significant.

**Figure 1:**
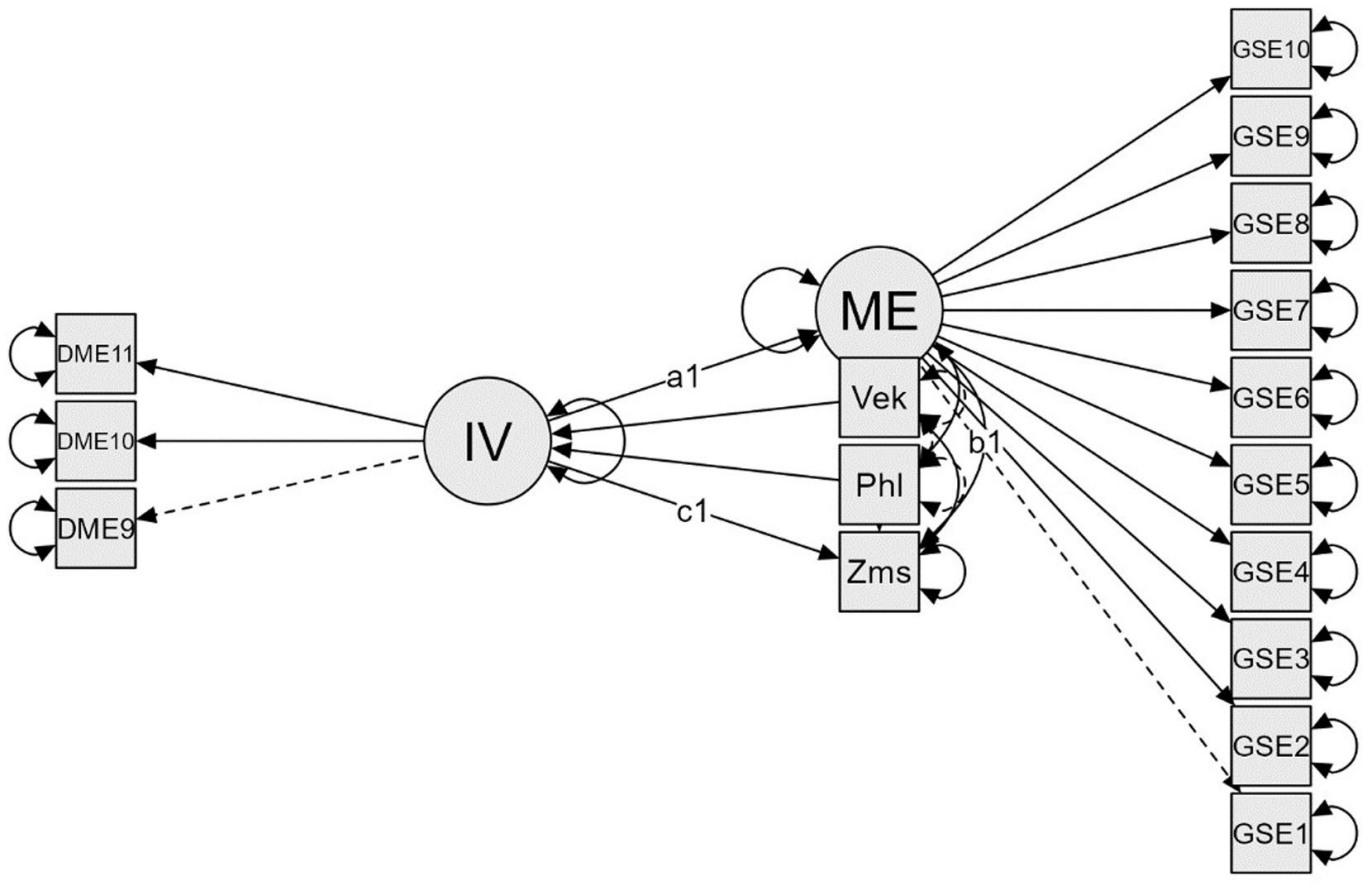
Structural model of mediation analysis. IV, career motivation; ME, generalized self-efficacy; Vek, age; Phl, gender; Zms, number of employers.

**Figure 2:**
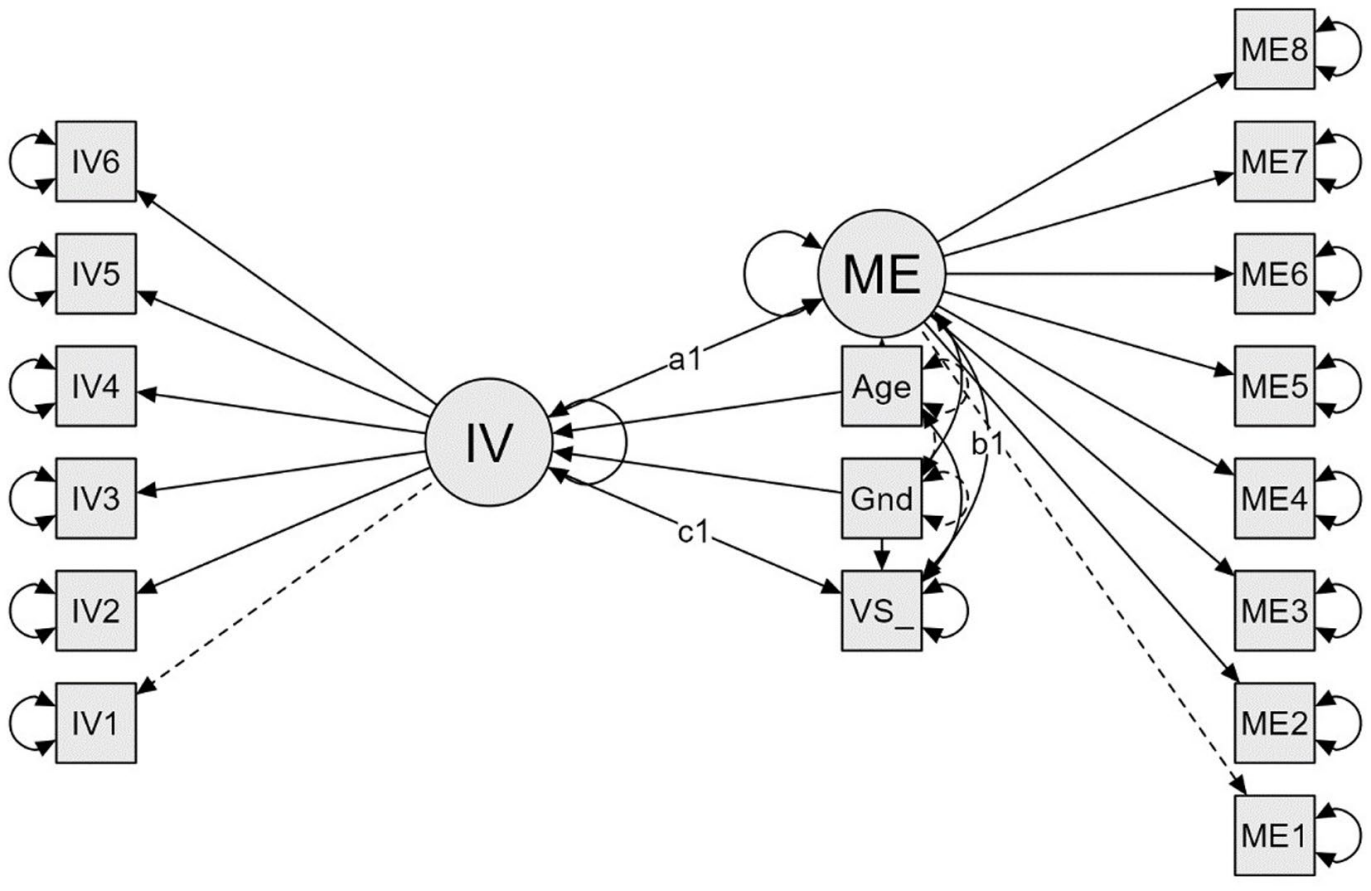
Structural model of mediation analysis. IV, task value; ME, time and study environment; VS, academic performance; Gnd, gender.

Based on the above findings, we hypothesize H1 that career motivation will predict the employability of university students, while this relationship will be mediated by generalized self-efficacy. Figure 1 expresses this relationship. We also expect the supporting hypothesis H2. Task value (the belief that study content is important, interesting, and useful), predicts academic achievement among higher education students and the organization of time and study environment mediates this prediction. Figure 2 illustrates the mediation model.

## 5. Methods

### 5.1. Participants

#### 5.1.1. Research sample 1

Three hundred and thirteen participants (72% women), with a mean age of 22.8 years (SD = 3,1), participated in the study. The research sample was selected by convenient sampling. The participant had to meet the condition of being a university student on the territory of the Slovak Republic. The age of the participants ranged from 19 to 41 years. The mean age of the women was *M* = 22.74; SD = 3.188 and that of the men *M* = 23.00; SD = 2.787. The study levels of the participants were represented as follows: First-level university education, bachelor’s degree (47%); Higher education second degree, master’s degree (40%); Higher education third degree, doctoral studies (13%). 92% of the participants reported belonging to the majority and 8% to the minority population. The representation of the study fields was as follows: Biology (20%); Ecology (6%); Economics (6%); Physics (7%); Marketing (5%); Medicine (20%); Pedagogy (10%); Law (9%); Psychology (10%) and Sport (6%). One hundred and fifty-six participants (50%) were currently employed. Of the currently working participants, it turned out that 46% of them work in their field and the rest are not employed in their studied field. Two hundred and eighty-one participants (90%) already had work experience.

#### 5.1.2. Research sample 2

The participants in this study were the convenience sample of 219 undergraduate students enrolled in nine higher education institutions in Slovakia. Sixty percent (*n* = 132) were women and 40 % (*n* = 87) were men. Their mean age was 22.98 years (SD = 3.72). Fifty-six per cent (*n* = 122) of the participants studied social sciences and humanities and 44 % (*n* = 97) of the participants studied life, health care or technical sciences. Participants were recruited *via* social media; data collection was voluntary and anonymous, with no reward for participation. Data were collected online during the second wave of the COVID-19 pandemic, students were experiencing distance or hybrid university education.

### 5.2. The research ethics

Before filling in the online questionnaire, participants agreed to participate in the investigation. Participation in the research was aligned with the principles of minimal risk (the risks anticipated in the research should not be greater than those commonly encountered in daily life), informed consent (participants were informed about any aspects of the study that could influence their willingness to cooperate; they could enter the study voluntarily and be allowed to withdraw from it at any time they desire without penalty), and right to privacy (information about a person acquired during a study must be kept confidential and not made available to others without the consent of the researcher).

Data were collected through an online survey with informed consent. The survey was anonymous and the participants were treated according to the ethical standards of the APA and the Declaration of Helsinki. This data collection was approved by the Ethics Committee of the Faculty of Social and Economic Sciences of Comenius University in Bratislava as part of the research project VEGA 1/0119/21.

### 5.3. Measurements

*Career Motivation* was measured by the dispositional measure of the employability subscale called career motivation (Fugate and Kinicki, 2008). It includes three items, e.g., “I have a specific plan for achieving my career goal” (Cronbach’s *α* = 0.689; McDonald’s *ω* = 0.698). Because the research sample consisted of university students, we added ‘university’ to the elements. Therefore, the original element, e.g., ‘I feel I am a valuable employee at work’, was expanded to: ‘I feel I am a valuable employee/student at work/university. The Slovak translation of the items was used, provided, and translated by several university workers based on the principle of consensus in the case of discrepancies. The participants rated the extent to which they agreed with the items on a 5-point Likert scale from 1 to 5, where: 1 – I do not agree at all 2 – I do not agree 3 – I have no strong opinion 4 – I agree 5 – I completely agree.

*The Generalized Self-Efficacy Scale* is an independent cultural questionnaire in 25 countries worldwide (Scholz et al., 2002; Luszczynska et al., 2005). For research purposes, we used the Slovak version (Košč et al., 1993). It contains ten items in a four-point Likert format from (1) “not true” to (4) “the truth” (Cronbach’s *α* = 0.872; McDonald’s *ω* = 0.875).

*Employability* was measured as the number of past and recent employers in students. Students themselves referred to the number. They stated 0 to 13 employers, an average of 2 employers.

*The Motivated Strategies for Learning Questionnaire* (MSLQ; Pintrich et al., 1991) was designed to assess motivational orientation among university students and their use of different learning strategies. This questionnaire consists of two parts, a section on motivation (31 items) assessing students’ academic goals, values, and beliefs about one’s ability to succeed in the course and a learning strategies section (50 items) all of which relate to the use of various cognitive and metacognitive learning strategies. The motivation section contains 6 subscales and the section on learning strategies contains 9 subscales. For this study, we used scores from two subscales: Task value (6-item subscale from the motivation section) and time and study environment (8-item subscale from the study strategies section). The reliability of the subscales measured by Cronbach’s alpha and McDonald’s was satisfactory with *α* = 0.92 and *ω* = 0.92 for the task value and *α* = 0.70 and *ω* = 0.70 for time and study environment.

*The study Performance* subscale of the Academic Achievement Questionnaire (AAQ) (Křeménková and Novotný, 2020) was used to assess self-reported study achievement. The subscale consists of four questions about the grades in general studies and the previous semester. The weighted average of the subscale with min = 1 and max = 6 is a measure of self-reported academic performance.

### 5.4. Data analysis

We processed the data using Lavaan-based structural equation modeling (SEM) in JASP 0.16.3 (JASP Team, 2022). We performed descriptive analysis, reliability analysis, Pearson’s correlation analysis, and structural equation modeling. We calculated indirect effects using bias-corrected percentile bootstrap with 5000 replications, 95% confidence intervals, and DWLS estimator for ordinary variables. To minimize the bias of the common method, the structural models were controlled by gender and age. We conducted two separate models with different samples and measures, study 1 and study 2. The dependent variable in the first study represented the objective measure of employability, operationalized as a number of past and recent employers, referred by the students. The dependent variable in the second study was measured as the real academic achievement, reported by the students. Depended variables in both studies were analyzed as the observed variables. The independent and mediating variables were analyzed as latent, in both studies.

## 6. Results

### 6.1. Hypothesis 1

The variables analyzed were correlated with a small to moderate effect size (Table 1). Correlations were controlled by age and sex.

**Table 1:** Means, standard deviations, and Pearson’s partial correlations.

Variable	*M*	*SD*	1	2	3
1. Employability	2.82	1.95	–		
2. Generalized self-efficacy	3.01	0.52	0.29***	–	
3. Career motivation	3.28	1.08	0.15**	0.30***	–

Conditioned on variables: Gender, age.

**p < 0.01, ***p < 0.001.

The structural equation model (Figure 1) showed a good data fit for the mediation model: *χ*^2^ = 61.584(97), *p* = 0.998, CFI = 1.000, TLI = 1.016, RMSEA = 0.000, SRMR = 0.040.

The indirect [95% CI (0.119.432)] and total [95%CI (0.032 0.614)] effects of the mediation analysis were significant. Because the direct effect was not significant [95%CI (−0.254 0.392)], the results show full mediation (Table 2). The identified mediator is aligned with the supposed model.

**Table 2:** Regression coefficients and parameter estimates.

Predictor	Outcome		Estimate	Std. Error	*z*-value	*p*	95% CI	
							*LL*	*UL*
Gender	Career motivation (IV)		0.291	0.104	2.785	0.005	−0.005	0.583
Age	Career motivation (IV)		0.069	0.015	4.678	<0.001	0.027	0.118
Self-efficacy (ME)	Employability (DV)	b1	1.336	0.230	5.796	<0.001	0.736	2.038
Career motivation (IV)	Employability (DV)	c1	0.092	0.143	0.643	0.520	−0.254	0.392
Gender	Employability (DV)		−0.731	0.236	−3.098	0.002	−1.185	−0.318
Age	Employability (DV)		0.116	0.045	2.604	0.009	0.049	0.182
Career motivation (IV)	Self-efficacy (ME)	a1	0.175	0.023	7.583	<0.001	0.107	0.267
Gender	Self-efficacy (ME)		0.132	0.032	4.141	<0.001	0.029	0.238
Age	Self-efficacy (ME)		0.008	0.005	1.650	0.099	−0.006	0.021
Indirect effect	a1 * b1		0.234	0.049	4.821	<0.001	0.119	0.432
Total effect	a1 * b1 + c1		0.326	0.123	2.643	0.008	0.032	0.614

CI, confidence interval; LL, lower limit; UL, upper limit; IV, independent variable; DV, dependent variable; ME, mediator.

### 6.2. Hypothesis 2

The variables analyzed were correlated with a moderate to large effect size (Table 3). Correlations were controlled for age and gender.

**Table 3:** Means, standard deviations, and Pearson’s partial correlations.

Variable	*M*	*SD*	1	2	3
1. Academic performance	4.74	0.83	–		
2. Task value	3.07	1.43	−0.409***	–	
3. Time and study environment	3.21	1.08	−0.494***	0.647***	–

Conditioned on variables: Gender, Age.

*** *p* < 0.001.

The model of the mediation equation model (Figure 2) showed an acceptable data fit: *χ*^2^ = 154.040 (112), *p* = 0.005, CFI = 0.985, TLI = 0.982, RMSEA = 0.042, SRMR = 0.071. Indirect effects [95% CI (−0.751, –0.240)] and total [95%CI (−0.432, –0.199)] of mediation analysis were significant. As the direct effect was not significant [95%CI (−0.099, 0.416)], the results show the full mediation (Table 4). The identified mediator is aligned with the supposed model.

**Table 4:** Regression coefficients and parameter estimates.

Predictor	Outcome		Estimate	Std. Error	*z*-value	*p*	95% CI	
							*LL*	*UL*
Gender	Task value (IV)		0.049	0.078	0.625	0.532	−0.286	0.384
Age	Task value (IV)		−0.021	0.008	−2.553	0.011	−0.058	0.010
Time and study environment (ME)	Academic performance (DV)	b1	−0.502	0.166	−3.030	0.002	−0.822	−0.269
Task value (IV)	Academic performance (DV)	c1	0.118	0.157	0.754	0.451	−0.099	0.416
Gender	Academic performance (DV)		0.156	0.140	1.114	0.265	−0.048	0.353
Age	Academic performance (DV)		0.001	0.015	0.079	0.937	−0.026	0.029
Task Value (IV)	Time and study environment (ME)	a1	0.853	0.069	12.314	<0.001	0.626	1.119
Gender	Time and study environment (ME)		−0.260	0.127	−2.037	0.042	−0.560	−0.010
Age	Time and study environment (ME)		0.004	0.015	0.278	0.781	−0.024	0.039
Indirect effect	a1 * b1		−0.428	0.147	−2.920	0.004	−0.751	−0.240
Total effect	a1 * b1 + c1		−0.310	0.032	−9.792	<0.001	−0.432	−0.199

CI, confidence interval; LL, lower limit; UL, upper limit; IV, independent variable; DV, dependent variable; ME, mediator.

## 7. Discussion

H1 has been supported. Generalized self-efficacy mediated the prediction of students’ employability. This prediction expresses full mediation, which means that generalized self-efficacy as an identified mediator is consistent with the assumed model (Figure 1). The results agree with the knowledge, that self-efficacy is the significant mediator in predicting employability (Liu et al., 2020; Magagula et al., 2020; Zhong et al., 2020; Zhao et al., 2021). The original contribution lies in the fact, that we measured employability as the number of real employers, not just the perceived self-assessment scale. The original contribution lies also in connecting career motivation and self-efficacy among other types of motivation (Wo et al., 2016; Tentama and Arridha, 2020). Based on the results and the previous ones, motivation seems to be the general predictor of employability, when mediated by self-efficacy. The results further confirm the importance of taking into account innate dispositions (here it was career motivation) in defining the goals that one wants to achieve and in a career decision-making (Larson and Borgen, 2006). We agree with the findings of Stajkovic et al. (2018) that individual differences in traits (career motivation) are more effective in achieving performance with the active participation of social cognition (generalized self-efficacy). Dynamic feature of personality, like the generalized self-efficacy, enables one to set the steps to the chosen goal, organize and plan them better.

H2 has been supported. The linear regression model in Study 2 confirmed the prediction of the academic achievement of higher education students by task value as a motivational aspect of their studies and with the organization of time and study environment as a mediator. Similarly to the model of H1, the prediction expresses complete mediation, which means that the organization of time and study environment was identified as the mediator, which is consistent with the proposed theoretical model (Figure 2). The findings are consistent with previous research showing the positive relationship between task value and academic achievement (Liem et al., 2008; Li et al., 2021) and the importance of the mediation effect of organizational time and study environment (Bong, 2001; Neuville et al., 2007). Although study success is often related to motivational constructs and beliefs (Steinmayr and Spinath, 2009; Stegers-Jager et al., 2012; van der Zanden et al., 2018; Steinmayr et al., 2019), it seems that organizational behaviors and time management play an important role in mediating this effect. Many researchers highlighted the importance of the organization of time and the study environment (e.g., West and Sadoski, 2011; Basila, 2014; Broadbent, 2017), and these findings appeared to be highly prevalent in the context of distance or online education. Higher education students have currently faced many barriers and challenges in effective studies due to the COVID-19 pandemic restrictions (Marler et al., 2021). Motivational beliefs mediated by effective study strategies (including organizational behavior and time management) appear to help them overcome these difficulties.

The results support the importance of integrating the dispositional (motivation) and dynamic social-cognitive characteristics (planning and organizing) of university students’ personalities on their way to reaching their goals (employability or academic achievement). Knowledge of motivation could be essential for the choice of goals. For example, people differ in their orientation to achievement (Schuler and Prochaska, 2011). People with lower achievement needs could profit from the social-cognitive approach (planning, and organizing skills) when reaching their goals. They could also focus their career on the less achieving environment, like a career in non-profit organizations, or helping professions. New results on collective efficacy could offer an option how to increase the individual level of self-efficacy (Veiskarami et al., 2017), and their planning and organizing skills.

The results of both presented models show that the success of higher university students operationalized as employability and academic achievement is predicted by motivational variables (career motivation and task value) with the mediation of their ability to regulate their effort and plan their studies and careers (generalized self-efficacy and organization of time and study environment). Motivation is crucial for defining goals. But on the way to the goal, it is not enough to be motivated, because the knowledge and skills how to get it mediate the path to success. This implies the opposite, that not motivated individuals will not utilize the knowledge of how to plan/organize steps toward the goal.

### 7.1. Practical implications

The current study results offer some suggestions for applied research in the field. Due to the motivational antecedent of self-efficacy, applied research could focus on the right way of developing individual self-efficacy, and thus planning and organizing skills. We suggest exploring how to choose the goals of performance concerning individual motivation or what kind of employer is suitable and more comfortable for an individual. The goals chosen should reflect the motivational conditionality of the personality. Strategies for the development of self-efficacy could depend on the nature of the personality. For example, people with low achievement motivation may look for goals/performances that bring them, above all, joy and fulfillment, because they will not be able to rely on the driving force of the desire for success. In addition, teachers could provide positive oral encouragement to support students’ self-efficacy and thus show positive attitudes that help motivate students in learning contexts (Dogan, 2015).

The relationship between task value, the organization of time and study environment, and academic performance highlights the importance of supporting university students in developing their motivation and time management and organizational study strategies. This seems to be highly relevant, especially in the period of transition from secondary to tertiary education (Watson et al., 2004) when students need to cope with a new system of education and requirements that may differ from those at the secondary level of their education. Based on the meta-analysis of 49 studies with more than 5000 participants, self-regulated learning training programs have the potential to enhance the academic performance of higher education students (Theobald, 2021). Higher education teachers may also implement the development of effective study skills in their courses and they should encourage students to set goals and monitor their performance (Lynch, 2010). The support of higher education students in self-regulated learning could also be beneficial to their ability to cope with academic stress and to improve overall academic well-being (Marler et al., 2021).

### 7.2. The limitation and future research implications

The limitation of current studies could lie in the research sample. The research participants were university students. The results could depend on the composition of the research sample. The samples were balanced for the fields of study; however, they were not balanced for gender. Some studies suggest the critical nature of gender in self-efficacy regression models (Saleem et al., 2011; Huszczo and Lee Endres, 2017). That is why, in the current studies, we controlled the regression models for gender.

Self-efficacy is currently the focus as the mediator in the relationship between performance and personality traits (Stajkovic et al., 2018). Some other moderator variables are in these analyses important too, e.g., work task complexity (Judge et al., 2007), extreme groups (Ambiel and Noronha, 2016); potentially traumatic events (Bosmans et al., 2015), or how we suggest the skill of planning and organizing the study. For further research, we recommend verifying relationships between self-efficacy constructs, performance, and attachment (Greškovičová and Hírešová, 2019; Klanduchová and Greškovičová, 2019). Skills such as self-awareness and adaptability are often considered predictors of employability (Bates et al., 2019; Bonesso et al., 2019; Cortellazzo et al., 2020; Römgens et al., 2020) and may serve as mediators/moderators in future research.

Self-regulated learning was the subject of many studies (Watson et al., 2004; Lynch, 2010; Credé and Phillips, 2011; Yip, 2012, 2021; Hilpert et al., 2013; Broadbent, 2017; Li et al., 2018), however, in the context of our results, we would suggest focusing further research on the motivation and learning strategies of vulnerable groups of higher education students and those who are at risk of early attrition, e.g., individuals with special educational needs (Antalová and Sokolová, 2022; Sokolová and Lemešová, 2022), mental health issues (Tinklin et al., 2005), or the first year students (Watson et al., 2004). Another important aspect of effective learning strategies at university is their sources, how much higher education students rely on strategies developed during their previous education, or how much effort they invest into developing new strategies after the transition to the university setting.

By applying the structural equation modeling with latent variables, we eliminated the measurement error (Baron and Kenny, 1986) and with dependent variables based on real-world evaluations (grades from teachers or number of employers), we could aspire to support a causal effect interpretation. At least, the current study can serve as the starting point for designing the experimental research, about the mediation effect of planning/organizing skills on the performance, predicted by motivation.

### 7.3. Contribution

Full mediation and nonsignificant direct effect supported the assumption, that for reaching the goals, it is not enough to be motivated or see the value in the task. It is crucial to know the steps and how to plan and organize them on the way to success (academic achievement and/or employability). Students need to combine their personality dispositions of motivation with learned strategies/skills for planning/organizing their study/employment. The importance of the mediator in form of planning/organizing skills was supported in two different samples of students. The data on students’ success were based on real criteria (real evaluations from teachers or the number of real employers). By supporting the full mediation in both studies, the importance of planning/organizing skills in combination with the motivation of students on their way to success was underlined. So, how to support motivated students? They can be educated *via* seminar assignments, expecting them to plan and organize their steps to the finalization of the task and providing feedback. Bruyere et al. (2022) showed that learning the strategy for applying the skills is crucial same as the content of the training.

## 8. Conclusion

The results of both studies support the hypothesis that the differences in higher-university students’ success are largely due to their motivation mediated by their ability to regulate their effort and plan their studies and careers. These findings are relevant not only for further research but also for planning career counseling and educational intervention for this particular generation of adolescents and young adults affected by changes in education due to the COVID-19 pandemic.

## Data availability statement

The raw data supporting the conclusions of this article will be made available by the authors, without undue reservation.

## Ethics statement

The studies involving human participants were reviewed and approved by Ethics Committee of Faculty of Social and Economic Sciences, Comenius University in Bratislava. The participants provided their written informed consent to participate in this study.

## Author contributions

All authors listed have made a substantial, direct, and intellectual contribution to the work and approved it for publication.

## Funding

This work was supported by VEGA 1/0119/21.

## Conflict of interest

The authors declare that the research was conducted in the absence of any commercial or financial relationships that could be construed as a potential conflict of interest.

## Publisher’s note

All claims expressed in this article are solely those of the authors and do not necessarily represent those of their affiliated organizations, or those of the publisher, the editors and the reviewers. Any product that may be evaluated in this article, or claim that may be made by its manufacturer, is not guaranteed or endorsed by the publisher.
